# Case-control analysis of LRRK2 protective variants in Essential Tremor

**DOI:** 10.1038/s41598-018-23711-w

**Published:** 2018-03-28

**Authors:** Adeline S. L. Ng, Ebonne Y. L. Ng, Yi Jayne Tan, Kumar M. Prakash, Wing Lok Au, Louis C. S. Tan, Eng-King Tan

**Affiliations:** 1 Department of Neurology, National Neuroscience Institute, Tan Tock Seng Hospital, 11 Jalan Tan Tock Seng, Singapore, 308433 Singapore; 2 Department of Neurology, National Neuroscience Institute, Singapore General Hospital, 20 College Road, Singapore, 169856 Singapore; 3 Neuroscience and Behavioural Disorders Program, Duke-NUS Medical School, 8 College Road, Singapore, 169857 Singapore

## Abstract

Co-existence of Parkinson’s disease (PD) and essential tremor (ET) may reflect overlapping pathophysiology underlying both conditions. Furthermore, PD patients with leucine-rich repeat kinase-2 (*LRRK2*) mutations may present with ET-like features, suggesting the possibility of common genetic underpinnings. Two common *LRRK2* variants, R1398H and N551K, have been shown to be protective in multiple PD cohorts. We hypothesized that R1398H and N551K may show a similar effect in ET. In a case-control study involving 3198 subjects (2680 controls and 518 ET cases), R1398H was detected in 16.6% of ET cases compared to 18.0% in controls (OR = 0.91, 95% CI = 0.71–1.17, p = 0.46); while N551K was detected in 16.5% of ET cases compared to 18.0% of controls (OR = 0.89, 95% CI = 0.69–1.15, p = 0.37). While these results suggest that *LRRK2* R1398H or N551K do not appear to modulate the risk of ET, it remains possible that a protective trend for both variants may be present in ET and a much larger sample size is required to identify this.

## Introduction

Essential tremor (ET) is a common movement disorder characterized mainly by postural and kinetic tremor of the upper extremities. ET and Parkinson’s disease (PD) are two of the most common movement disorders with overlapping clinical features^1^. Even though twin and family history studies show a high heritability for ET suggesting a strong genetic component for its pathophysiology, efforts to elucidate the genetic underpinnings of ET have only been moderately successful and the genetic determinants of ET remain unclear^2,3^. Mutations in the leucine-rich repeat kinase-2 (*LRRK2*) gene are the most common cause of familial PD, and PD patients with *LRRK2* mutations have been reported to present initially with an ET-like phenotype^4^, raising the possibility of a genetic link between PD and ET. Two polymorphic *LRRK2* variants rs7133914 (R1398H) and rs7308720 (N551K), both in linkage disequilibrium, were previously reported to confer up to 20% reduction in risk of PD in Chinese patients^5^, the protective effect of which has been replicated in other Asian cohorts^5,6^. Furthermore, the *LRRK2* N551K-R1398H-K1423K protective haplotype was also reported to have a frequency of >5% in a large Caucasian and Asian series^7^, with R1398H being the most likely functional variant and its protective effect appearing independent of other SNCA and microtubule-associated tau (MAPT) variants^8^. Given the possibility of shared genetic links between ET and PD, we conducted a case-control analysis to investigate if the apparent protective effect of the *LRRK2* R1398H and N551K variants would similarly be seen in our cohort of patients with ET.

## Results

DNA samples from a total of 3198 subjects comprising of 518 ET subjects and 2680 age- and race-matched controls were analysed. Overall, 90.4% were ethnic Chinese, while the rest were of mixed Asian ethnicity. The mean ± SD age of cases and controls was 51.4 ± 20.2 and 52.5 ± 11.5 years respectively, comprising approximately 54.8% men and 45.2% women altogether. The genotype frequencies of both *LRRK2* R1398H and N551K in ET and controls is summarized in Table 1. The genotype frequencies of heterozygous R1398H and N551K carriers was not significantly different in ET compared to controls. Five out of 518 ET cases and 21 out of 2680 controls were homozygous for the R1398H variant, while 6 out of 518 ET cases and 22 out of 2680 controls were homozygous for the N551K variant. Genotype frequencies did not vary according to age at onset in ET cases.

**Table 1:** R1398H and N551K genotypes and frequencies in ET cases and controls.

Genotype	ET	Controls	Total
R1398H			
GG (WT)	432 (83.4%)	2199 (82.1%)	2631 (82.3%)
AG	81 (15.6%)	460 (17.2%)	541 (16.9%)
AA	5 (1.0%)	21 (0.8%)	26 (0.8%)
N551K			
CC (WT)	433 (83.6%)	2196 (81.9%)	2629 (82.2%)
CG	79 (15.3%)	462 (17.2%)	541 (16.9%)
GG	6 (1.2%)	22 (0.8%)	28 (0.9%)
**Total**	**518**	**2680**	**3198**

WT = wild-type.

## Discussion

An association between ET and PD beyond chance co-existence has been widely recognized. Patients with asymmetric, childhood-onset ET who later developed PD, had their PD-related rest tremor typically starting ipsilateral to the side of the more severe ET tremor^9^. Other overlapping clinical features between ET and PD include bradykinesia, rigidity, and gait disorders along with other non-motor features, making differentiating between both disorders clinically challenging early in the disease course^10^. Furthermore, several epidemiological studies have demonstrated an association between ET and PD greater than expected in the general population^11,12^, suggesting the possibility of overlapping molecular and/or genetic links underpinning both diseases.

Despite strong evidence for genetic links in ET with more than half of affected individuals having a positive family history, identifying genes in both monogenic and complex forms of ET has been challenging. Difficulties encountered by ET genetic studies may result from relatively lax diagnostic criteria, lack of biomarkers for ET with high phenocopy rates, and high locus heterogeneity^2^. Linkage studies conducted in families with ET have reported mutations in *ETM2* (essential tremor monogenetic locus 2) and (fused in sarcoma) *FUS*, while other studies have linked (leucine-rich repeat and Ig domain containing 1) *LINGO1, FUS* and teneurin transmembrane protein 4 (*TENM4*) with ET^3^. Two genome-wide association studies demonstrated association between ET and variants in *LINGO1* and (solute carrier family 1 member 2) *SLC1A2*, with a meta-analysis confirming the association of rs9652490 in *LINGO1* with ET^2^.

Since the discovery of *LRRK2* mutations as a major cause of autosomal dominant parkinsonism^13^, multiple studies have attempted to investigate the role of both rare and common *LRRK2* variants in the pathophysiology of PD and other neurodegenerative disorders^14–17^. Genetic studies investigating the link between ET and *LRRK2* have yielded conflicting results: the *LRKK2* I2012T, G2019S and I2020T variants have been found to be rare in Caucasians with ET^18^, while a comprehensive analysis of the *LRRK2* gene found no association of common and rarer *LRRK2* mutations or *LRRK2* single nucleotide polymorphisms (SNPs) in ET cases or autopsy brain samples^19^. The common *LRRK2* G2385R variant widely shown to be associated with a two-fold increased risk of PD in various Asian populations was not found to be a significant risk factor for ET in our population^20^. While conversely, a novel *LRRK2* Val2390Met mutation was reported in a group of patients with tremor-dominant parkinsonism (TDP) who underwent sequencing of the entire *LRRK2* coding region, a notable finding as some patients displayed a clinical phenotype resembling ET converting to PD^21^. Furthermore, our group previously demonstrated an association between the known PD risk variant *LRRK2* R1628P with ET, where R1628P carriers had a two-fold risk of developing ET (OR = 2.20, 95% CI = 1.30–3.73, p = 0.0035)^22^.

Our group was the first to report the apparent protective effect of *LRRK2* variants N551K and R1398H in PD, which has since been replicated in other Asian PD cohorts^5,6^. Multivariate regression analysis suggest that R1398H and N551K confer a 20% reduction in PD risk independent of the Asian *LRRK2* risk variants G2385R and R1628P^5^. In non-PD cohorts, a non-significant protective trend was also observed for the *LRRK2* N551K-R1398H-K1423K haplotype in a clinical Lewy body dementia (DLB) series (OR 0.76, p = 0.061)^23^. Functional evidence for the protective effect of both variants come from *in vitro* studies using dopaminergic neuronal lines that revealed both G2385R and R1628P to display increased kinase activity in a toxic gain-of-function manner consistent with dominant missense *LRRK2* mutations, while the protective R1398H variant, on the other hand, displayed reduced extrinsic kinase activity^5^. Furthermore, *LRRK2* R1398H has been shown to increase GTPase domain dimerization and GTP hydrolysis, reduce active GTP-bound *LRRK2*, increase axon length of primary cortical neurones and enhance *LRRK2*′s stimulatory effect on the canonical Wnt signalling pathway, all in opposition to the effects seen with the *LRRK2* G2385R risk variant^24^. As N551K is in linkage disequilibrium with R1398H, it is possible that its protective effect is driven primarily by R1398H.

The results from this study show that the odds ratios (OR) for both variants appear to be higher in ET (R1398H OR = 0.91, 95% CI = 0.71–1.17, p = 0.46; N551K OR = 0.89, 95% CI = 0.69–1.15, p = 0.37) compared to PD^5^ (R1398H OR = 0.86, 95% CI = 0.77–0.97; N551K OR = 0.80, 95% CI = 0.69–0.93), suggesting that a protective trend for both *LRRK2* variants may be present in ET, but not high enough to reach statistical significance. It remains possible that these *LRRK2* variants exert a weaker effect in ET compared to PD, and a much larger sample size may be required to identify this. Nonetheless, to our knowledge, the size of our ET cohort in this study is one of the largest in current literature. Additionally, there remains a possibility that ET patients with R1398H or N551K variants may not develop PD (known as ET-onset PD, or ET-PD) due to the protective effect of both variants. Longer-term data on these ET carriers are needed to clarify this possibility.

While the results from this study do not preclude an effect of *LRRK2* in ET, we conclude that the *LRRK2* protective variants R1398H and N551K may not play a major role in modulating the risk of ET in our population. Further identification and study of rare *LRRK2* variants in ET is important, and would ideally be carried out in large multi-centre studies with sufficient power. Nevertheless, our current findings will potentially contribute to future pooled or meta-analyses of the role of *LRRK2* in ET.

## Methods

Consecutive ET cases recruited from the movement disorder clinics at the National Neuroscience Institute, and age-, gender- and race-matched controls were included this study. Some of the control subjects had participated in earlier studies^5,25^. ET was diagnosed clinically using the consensus statement of the Movement Disorder Society (MDS) on tremor^26^. The controls were not known to have any neurodegenerative diseases. The study received approval from the SingHealth Institutional Review Board ethics committee and all study subjects gave their informed consent. Genotyping of the *LRRK2* R1398H and N551K variants was carried out as previously described^5^. For a 20% effect size difference, our sample size has >80% power at alpha = 0.05. All methods were performed in accordance with the relevant guidelines and regulations. The authors agree to make materials, data and associated protocols promptly available to readers without undue qualifications in material transfer agreements.
